# Prevalence of Tracheoesophageal Anomaly Cases among Neonates Undergoing Surgery in a Tertiary Care Children’s Hospital

**DOI:** 10.31729/jnma.5242

**Published:** 2020-10-31

**Authors:** Bal Mukunda Basnet, Prashant Simkhada, Anupama Thapa Basnet, Raj Kumar Singh

**Affiliations:** 1 Department of Surgery, Kanti Children's Hospital, Maharajgunj, Kathmandu, Nepal

**Keywords:** *neonates*, *Nepal*, *tracheo-oesophageal anomalies*

## Abstract

**Introduction:**

Tracheo-oesophageal anomaly is the abnormal communication between trachea and oesophagus. The most common type of tracheo-oesophageal anomaly is oesophageal atreasia with distal tracheo oesophageal fistula. Tracheo-oesophageal anomaly is a common neonatal problem requiring an urgent surgery. Tracheo-oesophageal anomaly can be diagnosed as an isolated malformation or as part of polymalformative syndrome with possible vertebrae anomalies also known as Vacterl syndrome. The aim of the study is to find out the prevalence of tracheo-oesophageal anomaly cases among neonates undergoing surgery in a tertiary care childrens hospital in Nepal.

**Methods:**

This is a descriptive cross sectional study conducted in a tertiary care children's hospital in Nepal taking into account the medical records from period of 01 Jan, 2018 to Dec 31, 2019. Study population included the infants undergoing operative procedure in the operation theatre of Kanti Children's Hospital. The calculated sample size was 306. Data was collected by retrospective chart review technique and listed in performa. Consecutive sampling technique was used. Thus collected data was entered in SPSS version 20 and necessary calculations were done.

**Results:**

The prevalence of tracheo-oesophageal anomalies was found out to be 30 (9.8%) at 95% confidence interval. The most common problem, with which infants were brought to Kanti Children's Hospital, that required urgent neonatal surgical intervention was anorectal malformation constituting 94 (30.72%) of the surgeries followed by intestinal obstruction 76 (24.84%) which included duodenal atresia, jujunal atresia and ileal atresia.

**Conclusions:**

Tracheo-oesophageal anomaly constitutes a major portion of neonatal disease condition requiring surgery in Nepal.

## INTRODUCTION

Tracheo-oesophageal fistula (TOF) is the abnormal communication between trachea and oesophagus.^1^ Esophageal atresia (EA) is the most common congenital anomaly of the esophagus characterized by the complete discontinuity of the esophagus with or without an abnormal connection between the esophagus and the trachea.^2–4^ The survival rate is over 95%.^5^

EA-TOF can be diagnosed as an isolated malformation or as part of polymalformative syndrome with possible vertebrae anomalies (VACTERL syndrome).^6^ EA-TOF patients bear a higher risk of scoliosis during their lifetime.^7–9^ Operative techniques for the initial EA-TOF repair as well as the presence of vertebral malformation are two potential pathogenic mechanisms leading to development of scoliosis.^10–12^ The major causes of neonatal deaths are birth asphyxia, prematurity and severe infections.^13–14^ Surgical conditions remains as a major cause of neonatal admission to hospital.^15^

We attempt to find out the prevalence of teacheo-oesophageal anomaly cases among neonates undergoing surgery in Kanti Children's Hospital.

## METHODS

This is a descriptive cross sectional study conducted in a tertiary care children's hospital in Nepal taking into account the medical records from period of 01 Jan, 2018 to 31 Dec, 2019. The ethical approval was taken from the Institutional Review Committee of Kanti children's Hopsital (Ref: 633). Study population included the infants undergoing major operative procedure in the operation theatre of Kanti Children's Hospital. The calculated sample size should be 306. The sample size was calculated as follows:

$$\begin{array}{ll} \text{n} & \text{= }{\text{Z}}^{\text{2}}\times \text{p}\times \text{q}/{\text{e}}^{\text{2}}\\ & \text{= }{\left(1.96\right)}^{\text{2}}\times 0.1\times 0.9/{\left(0.05\right)}^{\text{2}}\\ & \text{= }3.84\times 0.09/0.0025\\ & =139 \end{array}$$

For consecutive sampling technique used, Sample size is doubled: 2Xn1

= 2 × 139

= 278

Considering 10% missing records:

278+10% × 278 = 306

Data was collected by retrospective chart review technique and listed in performa. Thus collected data was entered in SPSS version 20 and the descriptive statistical calculations were done.

## RESULTS

**Figure 1:**
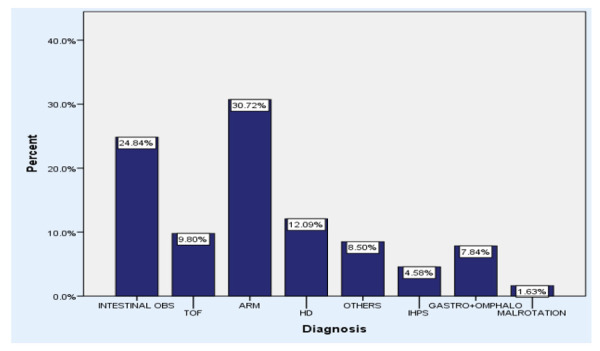
Prevalence of different neonatal conditions.

The prevalence of tracheo-oesophageal anomaly was found out to be 30 (9.8%) at confidence interval of 95%. The most common problem, with which infants were brought to Kanti Children's Hospital and required urgent neonatal surgical intervention was anorectal malformation constituting 94 (30.72%) of the surgeries followed by intestinal obstruction 76 (24.84%) which included duodenal atresia, jejunal atresia and ileal atresia. The frequency and percentage of various neonatal surgical procedures taking place in the Kanti Children's Hospital is shown (Figure 1).

**Figure 2:**
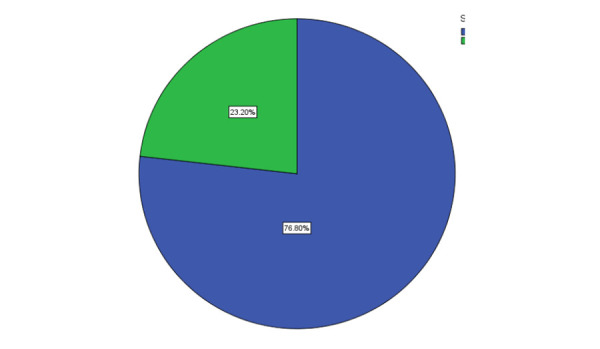
Male versus female.

Among those undergoing surgery, 235(76.8%) were male and 71 (23.2%) were females (Figure 2).

**Figure 3:**
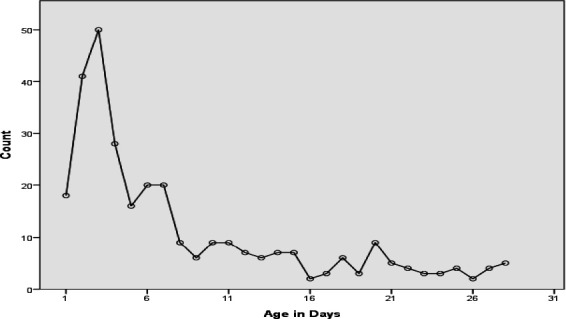
Presentation age in days.

Maximum of the neonates were brought to hospital by 3^rd^ day of life (Figure 3).

**Figure 4:**
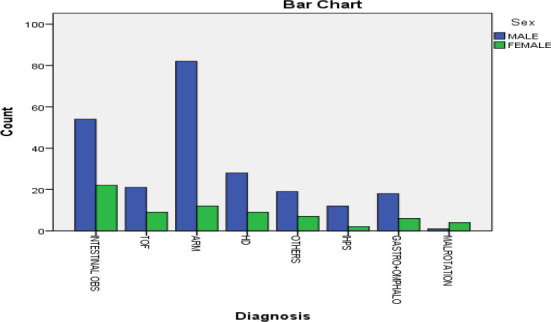
Individual disease prevalence by sex.

Male to female ratio for individual disease was highest for ARM (Figure 4).

## DISCUSSION

The prevalence of tracheo-oesophageal anomalies among neonates undergoing surgery in a tertiary care childrens hospital in Nepal was found out to be 30 (9.8%).

Among those undergoing surgery 235 (76.8%) were male and 71 (23.2%) were female. In a study by Abdul- Mumin A et al in Ghana, including 347 infants admitted to the NICU with neonatal surgical conditions, the number of male patients was 177 (52%) and female was 165 (48%).^16^ In a study done by Bastola R et al in Western Regional Hospital Nepal, the prevalence of congenital birth defect was found to be more in males.^17^

In their study, among the 1144 total admissions with congenital anoamaly 54.1 percentage of the patients were male which is lower compared to the percentage of males undergoing surgery in Kanti childrens Hospital, it is still higher compared to females presenting to their center.^17^

The most common problem was anorectal malformation followed by intestinal obstruction, Hirschprung's Disease, TOF and so on (Figure 1). In the study by Bastola R et al in Western Regional Hospital Nepal, maximun number of patient were admitted with congenital syndromes (Downs, Pirre Robins, Goldhenar's or Arthrogryposis multiplex congenita) followed by eye, ear, face and neck conditions followed by gastrointestinal malformations. In our study, gastrointestinal anomalies lead the table of causes of neonatal surgical procedure. This might be because of the surgical facility available at our set up and being a referral center from throughout the country. Similarly in the study by Abdul-Mumin A et al in Ghana ,the most common anomalies were omphalocele 48 (13.8%), imperforate anus 34 (9.8%), intestinal obstruction 29 (8.4%), spina bifida 26 (7.5%) and hydrocephalus 19 (5.5%).^16^

Maximum of the neonates were brought to hospital by 3^rd^ day of life. 193 (63.1%) of the neonates were less than 7 days by age.

The actual burden of the teacheo-oesophageal anomalies among the neonates of Nepal was unknown. In this study, we made an attempt to know the approximate of the condition in Nepal and also know the pattern of neonatal surgery in a tertiary care center in Nepal. For consecutive sampling technique sample size is doubled and sample size is adjusted considering 10% of missing records which avoids the selection bias, however being a single centered study it lacks the external validity.

## CONCLUSIONS

Tracheo- oesophageal anomaly constitutes a major part of neonatal disease condition requiring surgery in Nepal.
